# Co-Creating an Inclusion, Diversity, Equity, and Accessibility Strategy: Defining approach and outcomes in a health data research network

**DOI:** 10.23889/ijpds.v9i5.2856

**Published:** 2024-09-18

**Authors:** Amy Freier, Morgan Stirling, Nathan Nickel

**Affiliations:** 1 Health Data Research Network; 2 Manitoba Centre for Health Policy; 3 University of Manitoba

## Objective

Health Data Research Network Canada (HDRN) has committed to strengthening data use to improve health equity. Key this priority is has been an increased operationalization of Inclusion, Diversity, Equity, and Accessibility (IDEA) across the organization and within the data research processes HDRN Canada supports. Missing, however, was a unifying strategy for embedding IDEA within all HDRN Canada initiatives.

## Approach

Guided by results from an internal environmental scan and network feedback, HDRN Canada began developing an IDEA strategy in 2023. With the end goal of embedding IDEA across all internal teams and across all HDRN Canada strategic goals, collaborative participation methods were chosen to involve all levels of the organization. A process was designed that included focus groups, interviews, and roundtables.

## Results

Co-creating the IDEA strategy required broad buy-in including detailing expectations of the planning process in advance. A flexible management approach that enabled the team to adhere to the defined process allowed the Project Team to manage unanticipated challenges. 4 key strategies emerged focusing on learning, data quality and research, leadership, and community engagement. Developing the strategy with co-creative methods helped to identify practical approaches for HDRN to prioritize IDEA within its complex data research initiatives.

## Conclusion

HDRN Canada has outlined its commitment to IDEA in the health data ecosystem. The co-created strategy supports efforts towards accomplishing its organizational objectives and priorities. It is also a valuable resource for those in the health data space working to ensure data research contributes to equitable health outcomes.

